# Application and prospects of genetic engineering in CAR-NK cell therapy

**DOI:** 10.3389/fimmu.2025.1600411

**Published:** 2025-05-23

**Authors:** Caidong Hu, Wenhong Lai, Bin Tian, Xi Xu, Shuiling Xie, Wenting Zhong, Huiqiang Kang, Xiaoyun Chen, Hailiang Li, Jingxin Xu, Liping Liu

**Affiliations:** ^1^ Department of Hematology, Fujian Medical University, Fuzhou, China; ^2^ Department of Hematology, The First Affiliated Hospital of Gannan Medical University, Ganzhou, China; ^3^ Department of Neurology, Dongying Hospital of Traditional Chinese Medicine, Dongying, China

**Keywords:** natural killer cells, CAR-NK cells, genetic engineering, NK cell expansion, vectors for CAR expression, tumor microenvironment

## Abstract

With the rapid advancement of genetic engineering technologies, CAR-NK cell therapy, as an emerging immunotherapeutic approach, has demonstrated significant potential. CAR-NK cells recognize and eliminate tumor cells through chimeric antigen receptors (CARs). Genetic engineering techniques have enhanced the targeting and anti-tumor activity of CAR-NK cells by optimizing key components of the CAR structure, such as signal peptides, single-chain variable fragments (scFvs), linkers, and hinge regions. Additionally, NK cells can be derived from diverse sources, including peripheral blood, umbilical cord blood, stem cells, and NK cell lines, each with its unique advantages and limitations. Although CAR-NK cell therapy has shown promising anti-tumor efficacy in preclinical studies, it still faces numerous challenges. In the future, further optimization of CAR-NK cell design through genetic engineering and overcoming the immunosuppressive tumor microenvironment will be crucial for enhancing its clinical application efficacy. This review will comprehensively discuss the current applications, technical challenges, and future directions of genetic engineering in CAR-NK cell therapy.

## Introduction

With the rapid advancement of modern medicine, genetic engineering, as a cutting-edge biotechnology, is bringing revolutionary breakthroughs to the treatment of human diseases (1, 2). Among the various fields of genetic engineering applications, CAR-NK cell therapy, as an emerging form of cellular immunotherapy, is gradually becoming a focal point of research due to its unique advantages and immense potential (3–7). This article will delve into the current applications, technical challenges, and future directions of genetic engineering in CAR-NK cell therapy, focusing on key terms such as CAR, NK cells, and genetic modification.

## Principles and optimization strategies of CAR-NK cell therapy

### Structure and function of CAR

Chimeric Antigen Receptor, is a genetically engineered receptor protein. Its structure typically consists of three main components: the extracellular antigen recognition domain, the transmembrane domain, and the intracellular signaling domain. The extracellular antigen recognition domain is usually composed of a single-chain variable fragment (scFv), which specifically recognizes and binds to antigens on the surface of tumor cells. The transmembrane domain serves to connect the extracellular and intracellular regions. The intracellular signaling domain generally includes the CD3ζ chain and co-stimulatory molecules (such as CD28, 4-1BB, etc.), which activate the cell’s cytotoxic function upon antigen recognition.

The choice of promoter is critical in CAR-NK cell engineering, as it directly influences transgene expression levels, persistence and safety, highlighting the need to carefully balance strong anti-tumor activity with minimal off-target effects. (**Table 1**).

**Table 1:** Comparison of promoter types in CAR-NK therapy:.

Promoter Type	Examples	Advantages	Disadvantages
Viral Promoters	CMV, MPSV	-Strong CAR expression -Enhances anti-tumor potency	-Risk of tonic signaling (exhaustion/CRS) -Epigenetic silencing (e.g., methylation)
Constitutive Promoters	EF1α, PGK	-Stable, long-term expression -Lower toxicity	-Moderate expression may limit efficacy -Leakage in non-target cells
Endogenous Promoters	NKp46, CD16	-High NK-cell specificity -Genomic compatibility (safer integration)	-Typically weaker expression -Complex engineering (e.g., CRISPR)
Inducible/Synthetic	Tet-On, HRE	-Tunable CAR expression (e.g., hypoxia/drug-induced) -Reduced off-target toxicity	-Higher complexity/cost -Efficiency depends on external triggers

### Viral promoters

Viral promoters, such as CMV (cytomegalovirus) and MPSV (myeloproliferative sarcoma virus), are commonly used in CAR-NK cells due to their strong promoter activity. These promoters can drive high levels of CAR gene expression, thereby enhancing the anti-tumor activity of CAR-NK cells. Studies have shown that the CMV promoter can achieve up to 10-fold higher expression levels compared to endogenous promoters, making it particularly suitable for applications requiring robust CAR expression (8). Similarly, the MPSV promoter, derived from the murine leukemia virus, has been shown to provide strong and consistent expression in hematopoietic cells, including NK cells, which is advantageous for CAR-NK cell therapies (9). However, high surface density of CAR molecules (often driven by strong promoters) can induce tonic signaling through clustering. This tonic signaling has been associated with increased cytotoxicity, premature exhaustion of CAR-NK cells, and the potential for cytokine release syndrome (CRS), a systemic inflammatory response that can be life-threatening (10). To mitigate these risks, researchers have explored the use of inducible promoters or promoter systems that allow for fine-tuned control of CAR density, such as tetracycline-inducible systems or hypoxia-responsive elements (HREs) (11). Additionally, the use of viral promoters may be influenced by the sequence of the scFv within the CAR structure. studies have demonstrated that the inclusion of specific scFv sequences can result in promoter silencing or reduced expression due to epigenetic modifications, such as DNA methylation or histone deacetylation (12). This highlights the importance of carefully designing the CAR construct and considering the interplay between the scFv sequence and the promoter used.

### Constitutive promoters

Constitutive promoters enable sustained and stable expression of the CAR gene within cells. Compared to viral promoters, constitutive promoters generally exhibit lower promoter activity, which helps mitigate adverse effects associated with high CAR expression. The EF1α (elongation factor 1α) promoter, a housekeeping gene involved in protein synthesis, drives moderate and stable gene expression across various cell types, including NK cells, making it ideal for CAR-NK therapies requiring sustained CAR expression (13). Similarly, the PGK (phosphoglycerate kinase) promoter, derived from the phosphoglycerate kinase gene, offers reliable and moderate transgene expression, particularly effective in hematopoietic cells and suitable for CAR-NK engineering (14, 15). Both promoters provide consistent expression without the fluctuations seen with viral promoters. However, lower levels of CAR expression may compromise the anti-tumor efficacy of CAR-NK cells. Therefore, when selecting constitutive promoters, it is essential to balance CAR expression levels with cellular functionality.

### Endogenous promoters

Endogenous promoters refer to promoter sequences naturally present in the genome of an organism. They bind to RNA polymerase and initiate the transcription of genes. Compared to exogenous promoters, endogenous promoters exhibit higher tissue specificity and regulatory precision, allowing for fine-tuned control of gene expression levels based on cell type and physiological state (16, 17). By selecting endogenous promoters specific to NK cells, CAR gene expression can be efficiently and specifically targeted to NK cells, avoiding nonspecific expression in other cell types and thereby reducing potential side effects (13, 18). Endogenous promoters are more compatible with the host genome, minimizing the risks of genomic instability and insertional mutations during gene integration. Leveraging the regulatory mechanisms of endogenous promoters, conditionally expressed CAR-NK cells can be designed to activate CAR gene expression only under specific physiological or pathological conditions, enabling precise therapeutic control (19).

Researchers have explored the use of endogenous promoters to optimize CAR gene expression. For instance, in one study, replacing the endogenous promoters of CD16 and DNAM-1 genes enhanced the cytotoxicity of CRISPR-engineered NK-92 cells (20). Additionally, CAR-NK cells utilizing endogenous promoters have demonstrated promising anti-tumor efficacy and safety in preclinical studies, laying the groundwork for future clinical applications.

### Signal peptides

Signal peptides are critical sequences that guide the localization and secretion of proteins within cells. Their application in CAR-NK cell therapy is of significant importance for enhancing the expression efficiency and functionality of CAR proteins. By carefully selecting and optimizing signal peptides, the expression efficiency and cellular function of CAR proteins can be significantly improved, thereby enhancing the therapeutic efficacy and application potential of CAR-NK cells (21, 22). Common signal peptides include the CD8a signal peptide and immunoglobulin heavy or light chain signal peptides (23). The selection of an appropriate signal peptide requires consideration of its compatibility with the CAR protein and its secretion efficiency in target cells. For example, the CD8a signal peptide has demonstrated high expression efficiency in primary NK cells (24).

Modifying the amino acid sequence of signal peptides can improve their binding affinity to CAR proteins and secretion efficiency. For instance, bioinformatics tools can be utilized to predict and screen for the optimal signal peptide tailored to a specific CAR protein (25). Furthermore, fusing signal peptides with other functional sequences, such as linkers, can enhance the stability and functionality of CAR proteins. For example, combining a signal peptide with a flexible linker (e.g., GGGGS) helps maintain the correct conformation of the CAR protein (26).

### Single-chain variable fragment

The scFv is a critical component of the CAR structure, responsible for recognizing and binding to specific antigens on the surface of tumor cells. The scFv is composed of the variable light chain (VL) and variable heavy chain (VH) regions of a monoclonal antibody, connected by a flexible linker peptide (e.g., GGGGS). The scFv forms the foundation for the targeted therapeutic function of CAR-NK cells (27, 28).

Through genetic engineering, the amino acid sequence of the scFv can be modified to enhance its affinity for target antigens. For example, directed evolution or computer-aided design methods can be employed to screen for high-affinity scFv variants (29). To improve specificity, scFvs can be designed and selected to target tumor-specific antigens, minimizing off-target effects on normal cells (30). Additionally, by linking multiple scFv fragments together, multi-target CAR-NK cells can be constructed, enabling the simultaneous recognition and elimination of tumor cells expressing different antigens (31). This multi-target strategy helps overcome tumor heterogeneity and antigen escape.

Early scFvs were primarily derived from murine monoclonal antibodies, which exhibited high affinity and specificity but carried the risk of eliciting immune responses in humans (32). To reduce immunogenicity, researchers have developed humanized monoclonal antibodies. Humanized scFvs retain the high affinity of their murine counterparts while minimizing immune reactions in humans. Fully human monoclonal antibodies, whose scFvs are entirely derived from human sequences, offer even lower immunogenicity and higher biocompatibility (33, 34).

However, due to the chimeric nature of CAR receptors, even humanized scFv constructs may still induce host anti-idiotypic immune responses. Fortunately, in the limited CAR-NK clinical trials conducted to date, no major side effects related to anti-CAR immune responses have been observed. Studies have shown that both murine and humanized scFv-derived CAR-NK cells exhibit potent cytotoxicity against antigen-expressing tumor cells *in vitro* and *in vivo (* 3, 35, 36).

### Linker region

The linker region in CAR design is increasingly recognized for its role in optimizing scFv functionality and stability. Recent advances highlight its impact on antigen-binding affinity, CAR expression, and signaling efficiency (28). Innovative designs, such as protease-resistant sequences and non-natural amino acids, enhance stability and reduce immunogenicity (37, 38). Structured motifs, like proline-rich or β-sheet-forming sequences, improve VH-VL domain orientation for better antigen recognition (39, 40).

A significant breakthrough is the development of tumor microenvironment (TME)-responsive linkers, which activate CAR-NK cells selectively in response to TME-specific cues like pH or enzymatic activity, minimizing off-target effects (41–43). Computational tools, including molecular dynamics and machine learning, are now used to predict and optimize linker sequences for specific scFv architectures, accelerating next-generation CAR development (44, 45). These innovations enhance CAR-NK cell efficacy, addressing challenges like antigen escape and TME suppression.

### Hinge region

The hinge region, located between the scFv and transmembrane domain, is crucial for CAR-NK cell function, providing flexibility for scFv to access tumor antigens. Recent research focuses on optimizing hinge design through genetic engineering to enhance efficacy and safety. Common hinges include CD8α, CD28, and IgG, each with unique properties (46).

The CD8α hinge, derived from the CD8 molecule on T cells, is structurally stable and widely used in primary NK cells and CAR-NK cell lines (23). The CD28 hinge, derived from the CD28 molecule on T cells, has the ability to promote CAR dimerization (47). Compared to the CD8α hinge, the CD28 hinge is more likely to induce CAR dimerization, thereby enhancing the activation of CAR-NK cells. However, this may also lead to more severe CRS (48).

The IgG hinge, typically composed of the Fc region or CH2/CH3 domains of IgG1 or IgG4, offers high flexibility and stability, making it adaptable to various antigen recognition requirements (49). Its structural flexibility gives it broad application potential in CAR-NK cells. Although the IgG hinge is more commonly used in CAR-T cells, it is increasingly gaining attention in CAR-NK research.

### Transmembrane domain

The TM is a critical region in the CAR structure that connects the extracellular domain to the intracellular signaling domain. It anchors the CAR to the cell membrane, ensuring stable surface expression, and plays a key role in signal transduction by transmitting extracellular antigen-binding signals to the intracellular domain, thereby activating the cytotoxic mechanisms of NK cells. In CAR-NK cells, commonly used transmembrane domains include CD3ζ, CD8, CD28, NKG2D, 2B4, and DNAM1 (47).

CD3ζ and CD8α TM domains are critical for anchoring CARs to the cell membrane. Engineering these domains improves receptor stability and reduces off-target interactions, particularly in CAR-T cells targeting hematologic malignancies (50). Modifications such as charge redistribution (e.g., substituting lysine residues) enhance CAR-NK infiltration into immunosuppressive tumor microenvironments, addressing challenges in solid tumor therapy (51, 52). CD28-derived TM domains with dimerization motifs amplify co-stimulatory signals but increase CRS risk. Hybrid designs combining CD28 and CD8α TM reduce IL-6 secretion by 45% while maintaining anti-tumor activity (53).

The NKG2D receptor, a key activating receptor on NK cells, forms homodimers through cysteine residues in its transmembrane region. This structural feature enables stable disulfide bonds, enhancing receptor stability and ligand-binding efficiency to stress-inducible ligands on tumor cells. Preclinical studies demonstrate that engineering cysteine residues into the NKG2D TM domain of CAR-NK cells increases cytotoxicity by 3.2-fold compared to conventional designs, particularly in solid tumors like colorectal cancer (54). The 2B4 (CD244) and DNAM1 (CD226) TM domains synergize with activating receptors to amplify NK cell effector functions. This synergy enhances CD107a degranulation and IFN-γ secretion, counteracting immunosuppressive factors like TGF-β in the tumor microenvironment (55).

### Intracellular signaling domain

**Figure 1:**
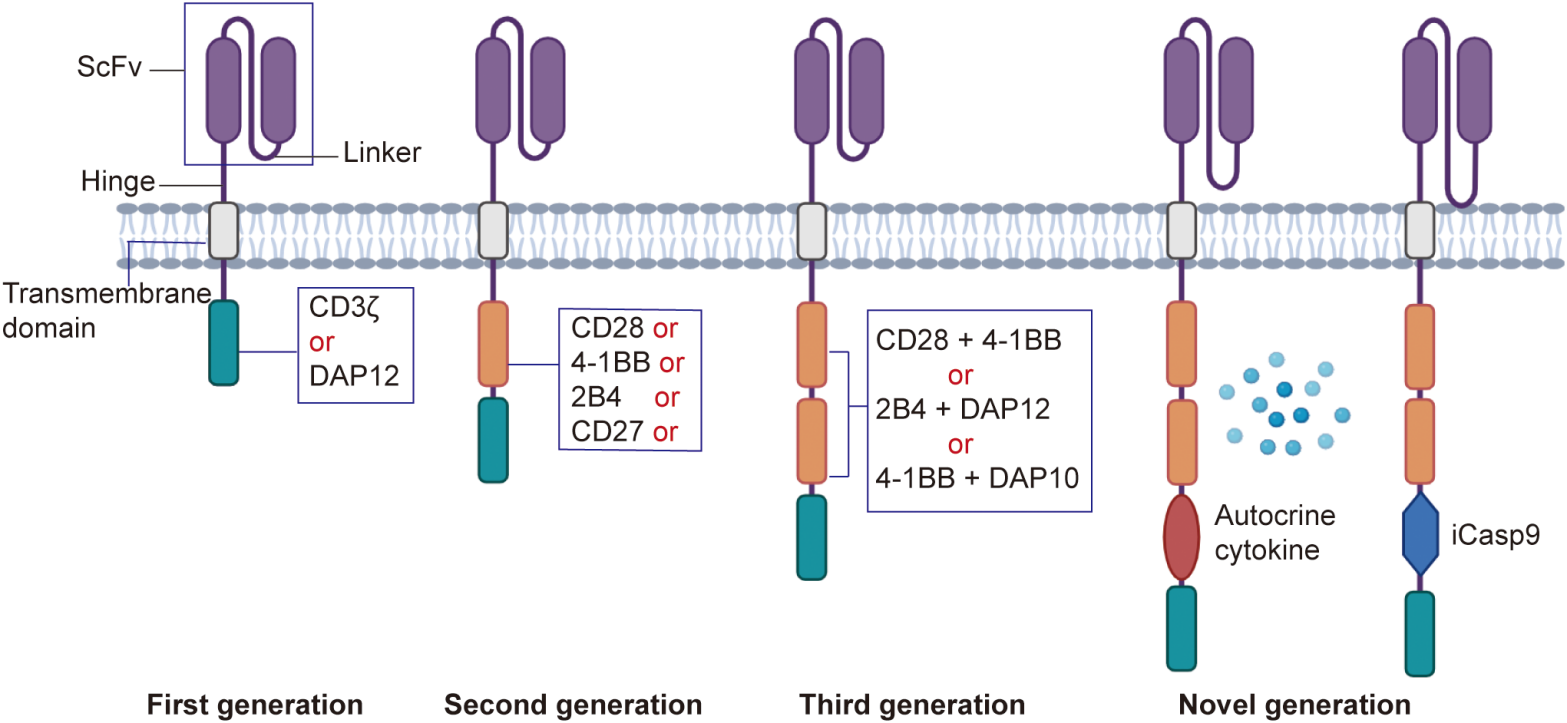
Development of the CAR structure. The first-generation CAR consists of an extracellular antigen-binding domain (scFv), a transmembrane domain, and an intracellular CD3ζ signaling domain, lacking co-stimulatory signals. The second-generation CAR incorporates one co-stimulatory molecule (e.g., CD28 or 4-1BB) in addition to CD3ζ, enhancing T cell activity. The third-generation CAR integrates two co-stimulatory molecules, further improving functionality. The next-generation CARs optimize safety, persistence, and targeting through gene editing, controllable switches, or multi-target designs, addressing complex therapeutic needs.

The intracellular signaling domain is responsible for transmitting activation signals to the NK cell upon antigen recognition (**Figure 1**).

- First generation CAR-NK cells contain only the CD3ζ signaling domain, which provides basic cell activation and cytotoxic functions. However, due to the lack of co-stimulatory signals, first-generation CAR-NK cells exhibit limited persistence and anti-tumor efficacy *in vivo (* 35, 56). DAP12 is an adaptor protein containing an immunoreceptor tyrosine-based activation motif (ITAM), capable of transmitting potent activation signals through its ITAM. In CAR-NK cells, DAP12 serves as an intracellular signaling domain, either replacing or complementing the traditional CD3ζ chain, thereby enhancing NK cell activation and cytotoxicity. Recent studies have demonstrated that the DAP12 signaling domain offers significant advantages in the treatment of both solid tumors and hematologic malignancies (57).
- Second generation CAR-NK cells incorporate at least one co-stimulatory domain, such as CD28 or 4-1BB, in addition to the CD3ζ domain. These co-stimulatory signals synergize with CD3ζ to enhance the activation and cytotoxicity of CAR-NK cells. CD28 signaling promotes cell proliferation and survival (58), while 4-1BB signaling improves cell persistence and memory formation (59). Other costimulatory domains, such as ICOS, OX40, CD27, and 2B4, have also been studied in T cells, demonstrating potential therapeutic benefits. Among these, 2B4, when combined with the DAP12 signaling domain, can synergistically enhance the functionality of CAR-NK cells (60).
- Third generation CAR-NK cells build upon the second generation by adding a second co-stimulatory domain, creating a dual co-stimulatory design. This further enhances anti-tumor activity and improves adaptability to the complex tumor microenvironment, thereby increasing therapeutic efficacy (61, 62).
- Novel generation CAR-NK cells retain the CD3ζ domain and incorporate multiple co-stimulatory domains, such as CD28, 4-1BB, and OX40 (63). To improve persistence, fourth-generation CAR-NK cells are engineered to express autocrine cytokines, such as IL-15, which plays a crucial role in NK cell development, survival, and activation. Autocrine IL-15 provides sustained growth factor support, prolonging the survival and enhancing the anti-tumor activity of CAR-NK cells *in vivo (* 64). Additionally, to enhance safety, fourth-generation CAR-NK cells integrate molecular safety switches, such as inducible caspase-9 (iCasp9) (65, 66). In the event of adverse effects or the need to terminate therapy, activating the safety switch can rapidly eliminate CAR-NK cells from the body, reducing treatment risks.

## Transfection or transduction vectors for CAR expression

### Viral vectors

**Figure 2:**
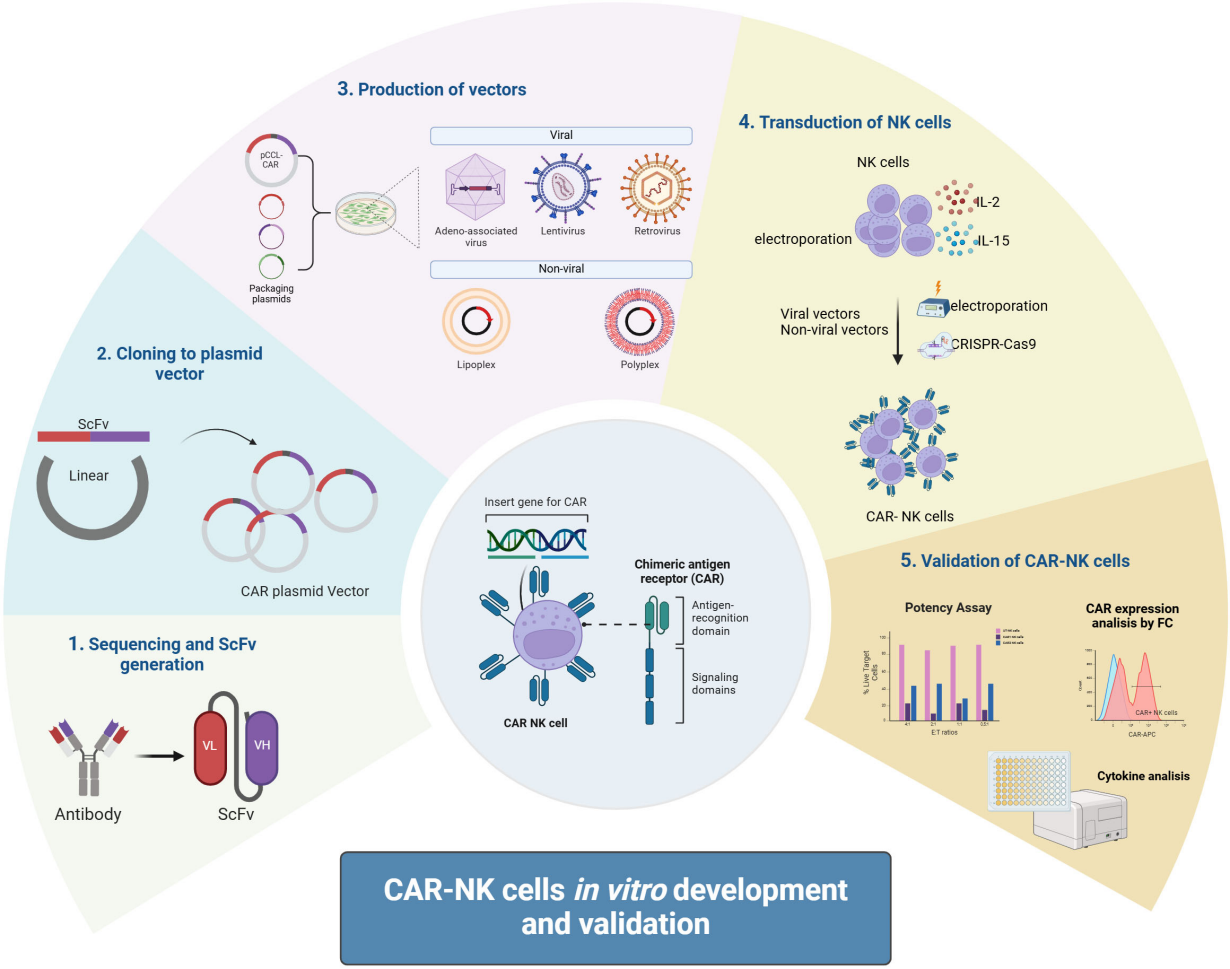
CAR-NK cells *in vitro* development and validation. CAR-NK cells are developed *in vitro* by first designing a CAR molecule targeting specific antigens. The CAR gene is then cloned into a plasmid vector, followed by packaging into a viral vector (e.g., lentivirus) or preparing mRNA. NK cells are transfected via viral transduction or electroporation, and validation includes assessing CAR expression, tumor cell cytotoxicity, cytokine secretion, and specificity through functional assays to ensure efficacy and safety.

Viral vectors are widely used in CAR-NK cell therapy due to their high-efficiency gene delivery capabilities (67). Commonly used viral vectors include lentiviral vectors and retroviral vectors (**Figure 2**).

- Lentiviral vectors exhibit broad cell tropism, enabling the infection of both dividing and non-dividing cells. Their genomes can integrate into the host cell genome, allowing for long-term and stable CAR expression. However, the random integration of viral vectors poses risks such as gene mutations and genotoxicity (68). Additionally, the transduction efficiency in primary NK cells is relatively low, often requiring multiple rounds of transduction. To improve transduction efficiency, researchers have developed specific vectors, such as baboon envelope pseudotyped lentiviral vectors (BaEV-LV) (69).
- Retroviral vectors, in comparison, have lower transduction efficiency and carry a risk of insertional mutagenesis, potentially leading to cell transformation (70). However, retroviral vectors preferentially infect actively replicating cells, and their transduction efficiency can be enhanced by stimulating NK cells and feeder cells with IL-2 prior to transduction (71). Furthermore, the production of viral vectors is subject to stringent regulatory requirements, increasing the complexity and cost of clinical applications.

### Non-viral vectors

Non-viral gene delivery systems, such as transposon systems (e.g., PiggyBac and Sleeping Beauty) (72, 73), utilize a “cut-and-paste” mechanism to efficiently transpose between vectors and chromosomes, resulting in stable CAR expression in CAR-NK cells. Compared to viral transduction, transposon systems offer higher gene transfer efficiency, lower levels of cell apoptosis, and reduced inter-individual variability (74).

- Electroporation and liposome transfection can effectively deliver exogenous genes into NK cells, with rapid gene expression and low levels of cell apoptosis (75). However, the exogenous DNA delivered by these methods does not integrate into the host genome, resulting in transient transgene expression.
- mRNA electroporation involves delivering mRNA into NK cells to express CAR proteins intracellularly. This method avoids the potential risks associated with genomic integration, and the transient nature of mRNA expression makes it suitable for scenarios requiring rapid generation of CAR-NK cells (76, 77). However, CAR expression typically lasts only a few days.

## Methods to enhance virus-mediated transduction in NK cells

### Optimization of viral vectors

Modifying the capsid proteins of viral vectors can enhance their binding affinity to receptors on NK cells, thereby improving transduction efficiency. For example:

- Mutations in the capsid proteins of adeno-associated virus (AAV) vectors can increase their ability to infect NK cells (78).
- Pseudotyping viral vectors with envelope proteins from different viruses (e.g., VSV-G, the glycoprotein of vesicular stomatitis virus) can broaden the host range and improve transduction efficiency in NK cells (79).

### Preconditioning NK cells

Preconditioning NK cells before transduction can enhance their activation, proliferation, and susceptibility to viral vectors:

- Cytokine pretreatment: Stimulating NK cells with cytokines such as IL-2 or IL-15 promotes their activation and proliferation, improving transduction efficiency (80).
- Physical stimulation: Techniques like electroporation can increase membrane permeability, facilitating the entry of viral vectors into NK cells (81).

### Optimization of transduction conditions

Adjusting transduction parameters can maximize efficiency while minimizing cell damage:

- Viral titer: Optimizing the viral titer based on NK cell count and state is crucial. Too low a titer may result in insufficient transduction, while too high a titer may cause cell damage or immune responses.
- Transduction time: Longer transduction periods generally improve efficiency but must be balanced against potential cell damage (82).
- Combination methods: Combining viral vectors with other gene delivery methods, such as electroporation, can significantly enhance transduction efficiency (83).

### Application of gene editing technologies

Gene editing tools like CRISPR/Cas9 can improve NK cell susceptibility to viral vectors:

- Knockout of inhibitory receptors: Deleting inhibitory receptors (e.g., NKG2A, CD96) enhances NK cell activation and proliferation, increasing their susceptibility to viral transduction (84).
- Enhancement of viral receptor expression: Upregulating genes related to viral vector infection (e.g., viral receptor genes) can improve transduction efficiency (85).

### Use of auxiliary systems

Additional strategies can further enhance transduction efficiency:

- Transduction enhancers: Chemicals like polyethyleneimine (PEI) or liposomes can increase membrane permeability or promote viral vector internalization, improving transduction efficiency (86).
- Co-culture systems: Culturing NK cells with feeder cells (e.g., irradiated PBMCs or K562 cells) provides a supportive microenvironment, enhancing NK cell activation and proliferation, and increasing their susceptibility to viral vectors (87).

### CRISPR/Cas9 gene editing technology

CRISPR/Cas9 gene editing technology offers powerful tools to optimize the functionality of CAR-NK cells through gene knockout, knock-in, and modification. For example:

- In primary NK cells, CRISPR/Cas9 has been used to disrupt the CD38 gene, preventing fratricide when NK cells are combined with daratumumab (an anti-CD38 therapy) (88).
- CRISPR/Cas9 can knockout inhibitory receptors on the surface of NK cells, enhancing the activity and anti-tumor capabilities of CAR-NK cells. For instance, knocking out CD96 and NKG2A significantly increases the cytotoxicity of NK cells (89, 90).
- The knockout of the TGFBR2 gene enables NK cells to counteract the immunosuppressive effects of transforming growth factor-beta (TGF-β) (91).

CRISPR/Cas9 technology allows for the precise integration of CAR genes into the NK cell genome, avoiding the risks associated with random integration by traditional viral vectors. For example, in some studies, CRISPR/Cas9-mediated homologous recombination templates (HDR) have been used to achieve efficient CAR gene knock-in in NK cells (92, 93).

Additionally, CRISPR/Cas9 can be combined with viral vectors to improve the efficiency and precision of gene editing. For instance, virus-like particles can deliver CRISPR components, enabling simultaneous CAR integration and gene knockout (94). CRISPR/Cas9 can also be paired with non-viral methods, such as electroporation, to introduce Cas9 protein and guide RNA into NK cells for targeted gene editing (95).

### Sources of NK cells

NK cells represent a promising platform for adoptive cell therapy, with diverse sources including peripheral blood, umbilical cord blood, stem cells, and immortalized cell lines, each offering unique advantages and challenges in terms of expansion potential, cytotoxicity, and clinical applicability. (**Table 2**).

**Table 2:** Comparison table of NK cell sources for CAR-NK therapy:.

NK Cell Source	Advantages	Disadvantages
PB-NK	-Mature phenotype with potent cytotoxicity -Clinically accessible (non-invasive collection) -Allogeneic use avoids GVHD (with T cell depletion)	-Limited abundance; requires extensive expansion -Functional exhaustion after prolonged culture -Heterogeneity and sensitivity to immunosuppressive TME
CB-NK	-High proliferative capacity -Ethically non-controversial, abundant source -Lower allogeneic rejection risk	-Reduced NK receptor expression after expansion -Immature phenotype may limit cytotoxicity
Stem cell-derived (iPSC/ESC)	-Unlimited scalable production -Low immunogenicity (autologous iPSC-NK) -Amenable to gene editing (e.g., CAR insertion)	-Immature functionality (weaker cytotoxicity/cytokine secretion) -Tumorigenicity risks (epigenetic memory, genetic modifications) -Ethical/legal concerns (ESC-derived)
NK cell lines (e.g., NK-92)	-Homogeneous, easy to expand (GMP-compatible) -High tumor sensitivity (KIR-negative) -Simple genetic engineering	-Aneuploid/malignant origin (requires irradiation) -No CD16 (limits ADCC unless engineered) -Short *in vivo* persistence post-irradiation

### Peripheral blood-derived NK cells

**Figure 3:**
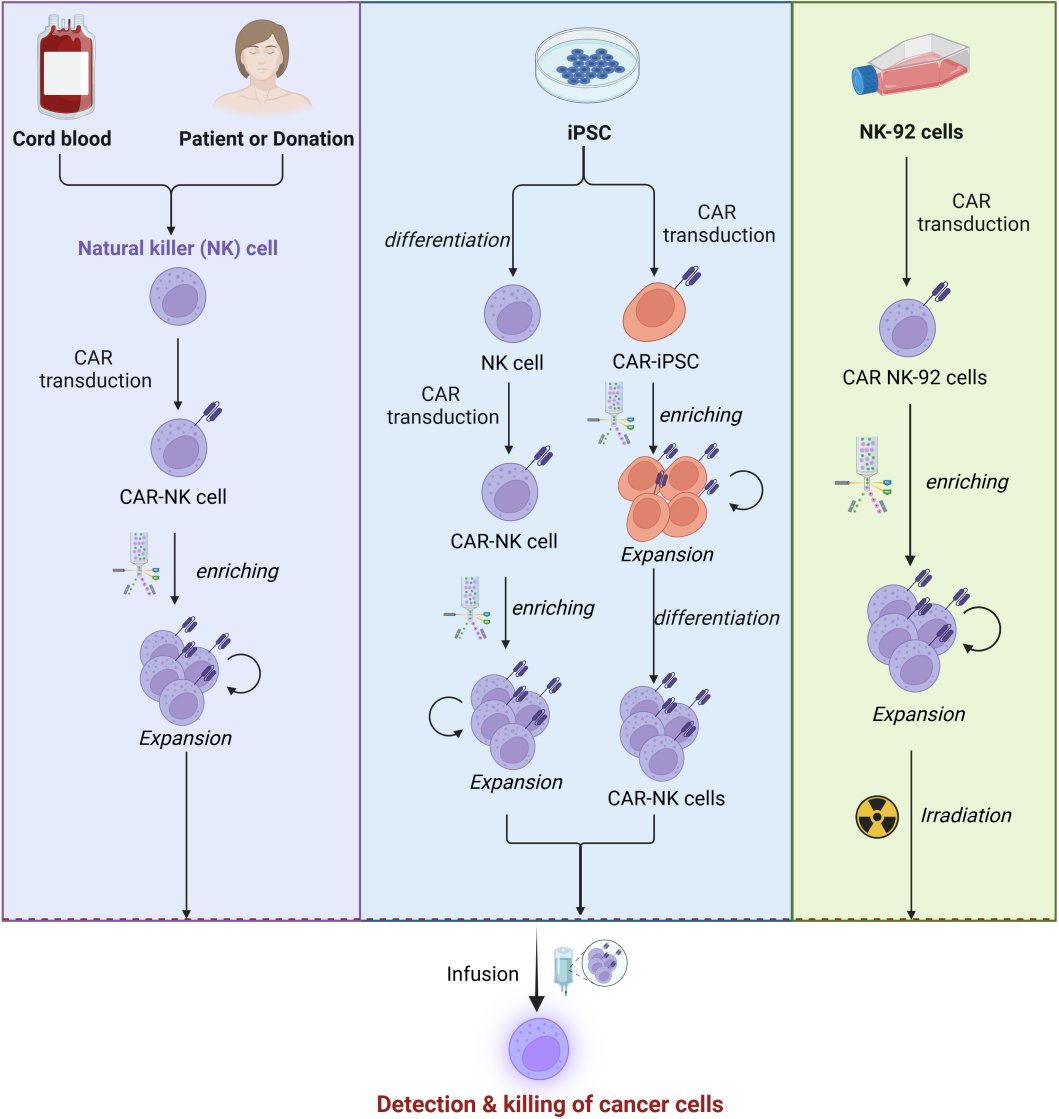
Sources of NK cells. CAR-NK cell manufacturing begins with sourcing NK cells from PBMCs, iPSCs, UCB, or NK-92 cells. These cells are isolated or differentiated and then transduced with a CAR construction, and enriched via magnetic-activated cell sorting (MACS) or fluorescence-activated cell sorting (FACS). Subsequently, the cells are expanded through co-culture with cytokines or feeder cells expressing IL-15 and mb-IL-21 to enhance proliferation and functionality. This process ensures the generation of potent CAR-NK cells for therapeutic applications.

PB-NK cells are isolated from whole blood or PBMCs using a non-invasive, clinically practical approach. Although autologous PB-NK cells may exhibit functional deficits due to patient-specific factors, allogeneic PB-NK cells are more commonly utilized in clinical settings, with T cell depletion being critical to avoid graft-versus-host disease (GVHD) (96) (**Figure 3**).

PB-NK cells are predominantly mature CD56dimCD16bright subsets, expressing activating receptors such as NKG2D, NKp44, and NKp46, which confer potent cytotoxicity against malignant cells (97). However, their limited abundance in peripheral blood necessitates extensive ex vivo expansion, typically achieved through cytokine cocktails (e.g., IL-2, IL-15, IL-21) or feeder cells like K562 (98, 99). Prolonged *in vitro* culture can lead to telomere shortening, reduced cytotoxicity, and functional exhaustion, driving ongoing research into optimized cytokine regimens and donor selection strategies to enhance expansion efficiency and functional persistence, particularly for CAR-NK cell applications.

Despite their therapeutic potential, challenges remain due to the inherent heterogeneity of PB-NK cells and the immunosuppressive tumor microenvironment. Factors such as regulatory T cells (Tregs) and tumor-derived immunosuppressive molecules can impair NK cell activity (100), limiting the consistency and efficacy of PB-NK cell-based therapies.

### Umbilical cord blood-derived NK cells

Umbilical cord blood-derived NK cells (CB-NK cells) have gained increasing attention in recent years due to their potent anti-tumor properties and unique advantages over conventional immunotherapies (101). Collected non-invasively from the umbilical cord and placenta during childbirth, cord blood provides an ethically acceptable and relatively abundant cell source without risk to the mother or infant.

CB-NK cells exhibit superior proliferative capacity compared to peripheral blood-derived NK cells (PB-NK cells). When expanded using feeder cells such as IL-21-expressing K562 cells, CB-NK cells demonstrate rapid proliferation and sustained cytotoxicity *in vitro (* 102). Clinically, a small cord blood unit can yield sufficient NK cells for therapeutic use within two weeks, with expanded CB-NK cells showing promising persistence and durability in trials. Furthermore, CB-NK cells are associated with a lower risk of immune rejection in allogeneic settings, enhancing their clinical safety profile.

Despite their advantages, CB-NK cells often show reduced expression of natural killer receptors during *in vitro* expansion, potentially limiting their cytotoxicity. Nevertheless, they retain strong tumoricidal activity against various malignancies, likely due to their heightened functional activity during early development. Advances in gene editing technologies have further enabled the enhancement of CB-NK cell anti-tumor efficacy, broadening their potential in cancer immunotherapy (103).

### Stem cell-derived NK cells

Advances in stem cell technology have enabled the generation of NK cells from various sources, such as embryonic stem cells (ESCs) and induced pluripotent stem cells (iPSCs), allowing for their expansion and functional optimization (104). While this technology offers numerous advantages, it also presents several challenges and potential drawbacks.

Stem cell-derived NK cells, particularly those derived from autologous sources like iPSCs, exhibit low immunogenicity, reducing the risk of post-transplant rejection and enhancing treatment safety. However, stem cell-derived NK cells may exhibit less mature cytotoxic and cytokine-secreting capabilities compared to other NK cell sources, potentially impacting their therapeutic efficacy.Gene editing and other technologies can be employed to enhance the functionality of stem cell-derived NK cells. For example, CAR-transduced iPSCs can differentiate into NK cells in the presence of specific cytokines. Similar to NK cells from other sources, iPSC-derived NK cells exert anti-tumor effects by secreting cytotoxic enzymes (e.g., perforin, granzymes), pro-inflammatory cytokines (e.g., IFN-γ, TNFα), or inducing apoptosis through direct cell contact mediated by TRAIL and Fas–FasL interactions (105, 106).

However, the long-term *in vivo* behavior of these cells remains unclear, with potential tumorigenicity risks, especially in genetically modified cells lacking thorough safety evaluations. Additionally, iPSCs may retain epigenetic memory from somatic cell origins, potentially altering lineage differentiation. Ethical and legal concerns also remain significant considerations in stem cell research, particularly when using embryonic stem cells, which may limit the broad applicability of this technology.

### NK cell lines

To date, several NK cell lines have been established, including NK-92, HANK-1, KHYG-1, NK-YS, NKG, NK101, NK3.3, YTS, and NKL (107, 108). These cell lines serve as excellent models for studying NK cell biology and related applications. Among them, NK-92 is the most extensively studied cell line in preclinical and clinical research. NK-92 cells are characterized by their homogeneity and ease of cultivation in large quantities, making them ideal for experimental and therapeutic purposes (56). They are particularly sensitive and robust in responding to tumor cells, largely due to their lack of killer immunoglobulin-like receptor (KIR) expression (109). Additionally, NK-92 cells can be easily genetically engineered under good manufacturing practice (GMP) conditions, highlighting their potential for industrial and clinical translation (62, 110, 111).

However, NK-92 cells are aneuploid and derived from malignant origins, necessitating irradiation before infusion to prevent uncontrolled proliferation. While irradiation ensures safety, it may limit the persistence of NK-92 cells *in vivo* and negatively impact their long-term therapeutic efficacy. Another limitation of NK-92 cells is their lack of CD16 receptor expression, which prevents antibody-dependent cellular cytotoxicity (ADCC). To address this, researchers have recently engineered high-affinity CD16 variants into NK-92 cells, enhancing their effector functions and broadening their therapeutic potential (112).

## *In vitro* NK cell expansion methods

### Cytokine stimulation

Cytokines play a critical role in the growth, activation, and expansion of NK cells:

- IL-2: A key cytokine for NK cell growth and activation, IL-2 promotes NK cell proliferation and enhances cytotoxic activity. It is commonly used in the expansion of CAR-NK cells.
- IL-15: More specific to NK cells than IL-2, IL-15 is often used as an alternative or in combination with IL-2 to improve expansion efficiency (64).
- Other cytokines: IL-21 and IFN-γ can also be used to further enhance NK cell activity, cytotoxicity, and functional maintenance during CAR-NK cell expansion (113, 114).

### Co-culture systems

Co-culturing CAR-NK cells with feeder cells or artificial matrices can enhance their growth and expansion:

- Feeder cells: Irradiated PBMCs or K562 cells are commonly used as feeder cells. They secrete various cytokines and growth factors that support the proliferation and expansion of CAR-NK cells (115).
- Artificial matrices: Materials such as fibronectin or laminin can mimic the *in vivo* cellular environment, promoting the adhesion and growth of CAR-NK cells (116).

### Genetic modification

Genetic engineering can optimize CAR-NK cell expansion and functionality:

- **IL-2 receptor enhancement**: Modifying CAR-NK cells to express high levels of IL-2 receptors (e.g., IL-2Rβ chain) increases their sensitivity to IL-2, enabling effective expansion even at lower cytokine concentrations (98).
- **Co-stimulatory molecules**: Incorporating co-stimulatory molecules (e.g., CD28, 4-1BB) into the CAR structure provides additional activation signals, promoting the expansion and functional maintenance of CAR-NK cells (117).

### *In vivo* CAR-NK research

The transformative potential of *in vivo* CAR-NK therapy lies in its ability to bypass the complexities of ex vivo genetic engineering through targeted delivery vectors that enable *in situ* gene editing. For instance, the CD8-targeted Nipah virus-pseudotyped lentiviral vectors (CD8-LVs) developed by the team of Christian J. Buchholz not only successfully generated CD19-specific CAR-T cells in humanized mouse models but also unexpectedly transduced CD8+ NK cells, which contributed synergistically to tumor clearance (118). The transformative potential of *in vivo* CAR-NK therapy lies in its ability to bypass the complexities of ex vivo genetic engineering through targeted delivery vectors that enable *in situ* gene editing. For instance, the CD8-targeted Nipah virus-pseudotyped lentiviral vectors (CD8-LVs) developed by the team of Christian J. Buchholz not only successfully generated CD19-specific CAR-T cells in humanized mouse models but also unexpectedly transduced CD8+ NK cells, which contributed synergistically to tumor clearance (119).

However, *in vivo* CAR-NK therapy faces several critical barriers. Target specificity remains a primary challenge: most current vectors (e.g., VSV-G-pseudotyped lentiviruses) rely on ubiquitously expressed receptors (e.g., LDLR), leading to nonspecific transduction (120). For instance, untargeted lentiviruses may infect off-target cells such as macrophages, which in the huSGM3 humanized mouse model significantly reduced CAR-T generation efficiency due to phagocytic clearance, a phenomenon likely to similarly impede CAR-NK cell programming (121). To address this, engineered shielded lentiviruses (CD8-LVsh) by modifying producer cells (e.g., β2M−/− CD47high HEK293T cells) were used to evade macrophage uptake, though further optimization is needed for NK cell transduction (122). Safety concerns also loom large: lentiviral genomic integration poses insertional mutagenesis risks, while AAVs, though safer, face preexisting immunity (about 50% of the population harbors neutralizing anti-AAV antibodies) that can compromise efficacy (123). Moreover, CD8-LV-induced CRS underscores the inflammatory risks of *in vivo*-generated CAR immune cells, a hazard equally relevant to CAR-NK therapies (118). Technically, the intrinsic of NK cells resistance to viral transduction results in low efficiency, and their short *in vivo* persistence necessitates adjunct strategies such as cytokine support or non-viral vectors. These challenges highlight the urgent need for NK-specific delivery systems, improved vector safety profiles, and refined functional control mechanisms.

## Technical challenges of genetic engineering in CAR-NK cell therapy

Gene transduction is a critical step in constructing CAR-NK cells, yet current methods face significant limitations. Viral vector-mediated transduction, while highly efficient, carries the risk of insertional mutagenesis, potentially leading to abnormal cell proliferation or malignant transformation. Studies have shown that lentiviral vector-induced p53 activation may result in impaired cellular proliferation, G1/S phase cell cycle blockade, and a modest yet statistically significant elevation in apoptotic rates during *in vitro* expansion (124). During autologous CAR-T production, accidental CAR gene transduction occurred in a leukemic B cell. This led to surface co-expression of the anti-CD19 scFv and CD19, enabling immune evasion from CAR-T recognition and therapy resistance (125). CRISPR-Cas9 genome editing can trigger p53-dependent DNA damage responses and cell cycle arrest in human cells (126). Additionally, pre-existing immunity against Cas9 (both antibody and T-cell responses) poses clinical risks for *in vivo* applications (127). Additional, non-viral methods, such as electroporation and lipid-based transfection, offer improved safety but suffer from lower transduction efficiency, making large-scale CAR-NK cell production challenging. Thus, balancing high transduction efficiency with safety remains a major hurdle in CAR-NK cell engineering.

The persistence and stability of CAR-NK cells *in vivo* directly impact therapeutic efficacy. Clinically, CAR-NK cells must survive long enough to exert sustained antitumor effects. However, NK cells have a relatively short lifespan and are susceptible to immunosuppressive factors *in vivo*, leading to gradual declines in activity and numbers (30). Additionally, CAR-NK cell stability is influenced by CAR structural design and gene expression regulation. Enhancing the *in vivo* persistence of CAR-NK cells while maintaining their stability and cytotoxic function through genetic engineering remains an unresolved challenge.

The TME presents a formidable challenge to CAR-NK cell therapy due to its highly immunosuppressive nature, characterized by the presence of Tregs, MDSCs, and TAMs that actively suppress CAR-NK cell function through multiple mechanisms, including the secretion of inhibitory cytokine like TGF-β and IL1-10, expression of immune checkpoint molecules such as PD-L1, and metabolic disruption via arginase and reactive oxygen species production (128). Furthermore, the TME’s acidic conditions, resulting from lactate accumulation, and elevated adenosine levels through A2A receptor signaling further impair CAR-NK cell cytotoxicity and persistence (129). The dense extracellular matrix (ECM) formed by cancer-associated fibroblasts creates physical barriers that hinder CAR-NK cell infiltration, while hypoxia-driven metabolic changes in tumor cells exacerbate these suppressive effects, collectively creating a hostile milieu that necessitates innovative genetic engineering strategies to enhance CAR-NK cell resistance and functionality within this complex ecosystem (130).

## Conclusions

CAR-NK cell therapy, an emerging immunotherapy, shows great potential in cancer treatment. Genetic engineering enhances CAR-NK cells by optimizing scFv, hinge, transmembrane, and signaling domains. NK cells from peripheral blood, cord blood, stem cells, or cell lines each have unique pros and cons. Despite promising preclinical results, challenges include *in vivo* persistence, tumor microenvironment suppression, and large-scale production. Balancing gene transduction efficiency with safety, ensuring functional efficacy within the complex tumor microenvironment, and addressing technical hurdles in large-scale production are critical issues that require further optimization through genetic engineering. Moving forward, integrating advanced gene editing technologies like CRISPR/Cas9 to design more efficient and safer CAR-NK cells will be pivotal for advancing their clinical applications.
